# The Effect of Reframing the Goals of Family Planning Programs from Limiting Fertility to Birth Spacing: Evidence from Pakistan

**DOI:** 10.1111/sifp.12155

**Published:** 2021-05-20

**Authors:** Saman Naz, Yubraj Acharya

**Affiliations:** ^1^ Saman Naz, Yubraj Acharya, Department of Health Policy and Administration College of Health and Human Development Pennsylvania State University USA

## Abstract

Contraceptive prevalence in Pakistan has plateaued near 34 percent for over a decade, suggesting that fertility levels are likely to stay high unless effective interventions are designed. We evaluate the Family Advancement for Life and Health 2007–2012 (FALAH), a family planning project implemented in 31 districts of Pakistan. Deviating from previous programs, FALAH emphasized birth spacing—as opposed to limiting family size—as the primary purpose of contraceptive use. We use Pakistan Demographic and Health Survey to evaluate FALAH's impact on continuous and binary measures of birth intervals. To estimate the causal effects of the project, we compare the outcomes for multiple children born to the same mother before and after the project. We find that FALAH increased interbirth intervals by 2.4 months on average and reduced the proportion of short birth intervals by approximately 7.1 percentage points. This finding suggests that birth spacing as a goal of contraceptive use may resonate better with Pakistani couples than limiting family size. The project's effects were more pronounced for women with high education, in rural areas, and in the middle of the wealth distribution.

## INTRODUCTION

Pakistan is the fifth most populous country in the world with 216.6 million inhabitants (PRB 2020). At 3.6, Pakistan's total fertility rate (TFR) is the highest in South Asia, with only a small decline during the past decade (from 4.1 in 2007 to 3.6 in 2018) (NIPS and ICF 2013, 2019). The most recent Pakistan Demographic and Health Survey (PDHS 2017–2018) shows that the contraceptive prevalence rate (CPR) *decreased* from 35.4 percent in 2013 to 34.2 percent in 2018, despite a high unmet need for contraception (17.3 percent) (NIPS and ICF 2019).

Reflecting the low CPR, the majority of the birth intervals in Pakistan—66 percent according to the PDHS 2017–2018—are shorter than the 36 months recommended by the World Health Organization (WHO 2005). Furthermore, 36 percent of children are born within 24 months of the previous birth, an interval perceived to be “too short” (NIPS and ICF 2013).

Pakistan is the only country in South Asia (except Afghanistan) where the fertility rate is still above replacement level (PRB 2020). The country did not meet Millennium Development Goals on maternal and child health (Rizvi et al. 2015). Existing literature from Pakistan points to a number of factors that may contribute to the lack of effectiveness of the family planning programs in reducing fertility. These factors include a lack of political support for the programs, insufficient financing, de‐motivated health workers, an inefficient health information system, a nonresponsive service delivery system, and a weak contraceptive supply chain system (Cleland et al. 2006; Mumtaz et al. 2013; Nishtar et al. 2013; Ali, Azmat, and Hamza 2018). On the demand side, previous studies have identified psychosocial factors including social conservatism, preferences for larger families and sons, and religion as reasons for low contraceptive uptake and the resulting high fertility (Agha 2010; Channon 2017; Sathar 2013). Some researchers have also criticized the narrow focus of the family planning programs on promoting small families rather than on addressing the aspirations, beliefs, and needs of couples regarding their families (Cleland et al. 2012; Mir and Shaikh 2013; Ataullahjan, Mumtaz, and Vallianatos 2019; Agha 2010), as such focus has led couples to believe that the state is imposing restrictions on their choices about family size (Mir and Shaikh 2013).

In 2007, a project called Family Advancement for Life and Health (FALAH) was initiated which deviated from the usual practice of promoting small family size as the goal of contraceptive use and repositioned family planning as a health intervention aimed at improving the health of mothers and children through adequate birth spacing (Capps et al. 2012). In this paper, we evaluate the project to assess if the repositioning of family planning as a health intervention was effective in increasing spacing between births.

Various aspects of FALAH have previously been examined, including its impact on unmet need (Jain et al. 2014), men's attitude towards family planning (Ashfaq and Sadiq 2015), contraceptive prevalence (Mahmood, 2012), and healthcare providers’ perception of birth spacing (Mir and Shaikh 2013). All of these studies point to generally positive effects of the project.

One of the key limitations of these studies is that they do not directly assess the project's effect on birth spacing, one of its main objectives. A second limitation is that, given their descriptive nature, the studies do not establish the causal link between the project and the outcomes they evaluate. Finally, the studies use administrative data from the project itself which are more prone to reporting bias than data such as PDHS that are collected independently.

As the first contribution of our study, we use an innovative empirical approach and address the limitations of the existing studies by attempting to causally link the intervention to its intended outcome (birth spacing). Our study thus complements the existing literature on FALAH. Second, we evaluate a large project from a setting where contraceptive use is low, birth intervals are narrow, and fertility has been difficult to reduce, despite decades of effort from the government and development agencies. Pakistan's experience from FALAH may inform policy design in other similar settings around the world, thus contributing to the Sustainable Development Goals on improving the health and well‐being of women and children.

To preview the results, we find that FALAH increased birth spacing by 2.4 months, which translates to an approximately 9.3 percent increase in birth intervals from the average. As such, the proportion of birth intervals deemed short by WHO fell by approximately 7.1 percentage points. These findings, which are robust to a range of checks, suggest that birth spacing as a goal of contraceptive use may resonate better with Pakistani couples than limiting family size. The project had heterogeneous effects based on mother's education, area of residence, and household wealth, with the effects more pronounced for those with high education, in rural areas, and in the middle of the wealth distribution.

The rest of the paper is organized in the following manner. In the second section, we provide a brief overview of the FALAH project. We discuss our data and the empirical strategy in the third and fourth sections, respectively. In the fifth section, we present our findings, including results from various robustness checks. We conclude in the sixth section with a discussion of the key limitations, policy implications, and areas for future research based on our findings.

## FAMILY ADVANCEMENT FOR LIFE AND HEALTH (FALAH) PROJECT

**FIGURE 1:**
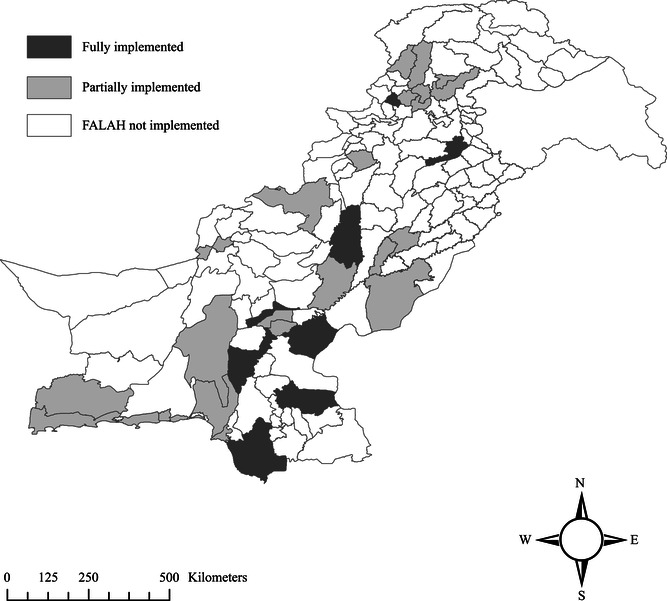
Map showing the districts where FALAH was implemented NOTES: This figure shows the districts where the FALAH project was implemented. In the following 10 districts the project was implemented for the entire duration of five years: Dera Ghazi Khan, Jhelum (Punjab province); Dadu, Ghotki, Larkana, Sanghar, Sukkur, Thatta (Sindh province); Charsadda (Khyber Pakhtunkwa province); Jaffarabad (Balochistan province). In another 21 districts, the project was implemented for a shorter duration due to various reasons including donor preferences and security issues in the region (Capps et al. 2012): Bahawalpur, Khanewal, Multan, Rajanpur (Punjab province); Jacobabad, Karachi, Shikarpur (Sindh province); Battagram, Buner, Lakki Marwat, Mansehra, Mardan, Swabi, Swat, Upper Dir (Khyber Pakhtunkwa province); Gwadar, Khuzdar, Lasbella, Quetta, Turbat, Zhob (Balochistan province).

The FALAH project was launched in 2007 with the objective of improving the well‐being of families through increased demand and utilization of birth spacing and quality family planning services (Mahmood 2012). The project was first implemented in 20 districts across four provinces of Pakistan. In July 2009, 11 additional districts were added and five were dropped due to security concerns (Capps et al. 2012). During the last year of the project, the activities were limited to only 15 districts (Capps et al. 2012). The project was therefore implemented for all five years in 10 districts and for fewer than five years in 21 districts (Figure 1). Overall, the FALAH project districts cover 37 percent of Pakistan's total population. The US Agency for International Development (USAID) funded the project. A consortium of NGOs led by the Population Council implemented it, in collaboration with provincial governments.

Like many family planning programs around the world, FALAH's full package of interventions included behavior change communication and measures to strengthen the health system along with community mobilization activities (Jain et al. 2014; Mahmood 2012; Mir and Shaikh 2013; Sathar 2013). However, one distinct feature of the project was its messaging. Behavior change communication interventions included a campaign called “Healthy Timing and Spacing of Pregnancy” (HTSP) conducted through mass media, community media, and interpersonal communication (Mahmood 2012; Jain et al. 2014; Mir and Shaikh 2013). As part of this campaign, the project aimed to shift the focus of family planning away from the traditional concept of promoting small families to a new concept that encouraged birth spacing as a way to improve maternal and newborn health (Mahmood 2012; Jain et al. 2014; Mir and Shaikh 2013). This was aimed at removing cultural and religious barriers to family planning and creating an overall acceptance of family planning in the society (Mir and Shaikh 2013). In this regard, the project formulated specific messages to address misgivings associated with the Islamic viewpoint on family planning and how birth spacing directly impacts maternal, child, and family health (Ashfaq and Sadiq 2015).

An estimated 48 million people heard FALAH's mass media messages on the benefits of birth spacing (Capps et al. 2012). The average viewer was exposed to FALAH advertising message approximately 48 times (Capps et al. 2012). Project monitoring documents show that those who saw or heard the FALAH media messages confirmed that “they saw a difference between birth spacing messages that highlight benefits for maternal and child health and the message from previous family planning campaigns that emphasized limiting the number of children. They also said that the new HTSP messages were more acceptable to them and that they would share what they had heard with others” (Capps et al. 2012, 18).

Messaging through interpersonal communication was directed in a similar manner. As part of the training provided by the community‐based health workers (locally called lady health workers ), women were told about the health benefits of birth spacing and how HTSP was consistent with Islamic beliefs. Project monitoring documents show that the beneficiaries of these training acknowledged that the new messages about birth spacing were new to them and that they appreciated the focus away from limiting family size (Capps et al. 2012). These activities reached approximately 5.7 million men and women in the project districts (Capps et al. 2012).

## DATA

We use birth history data from the Pakistan Demographic and Health Survey 2012–2013. The PDHS collected detailed information from married women between the ages of 15 and 49 years about their pregnancies and births (NIPS and ICF 2013). This nationally representative survey includes data on 13,558 currently married women and 50,238 births. We use the individual recode file, which includes all live births of the sampled women.

**FIGURE 2:**
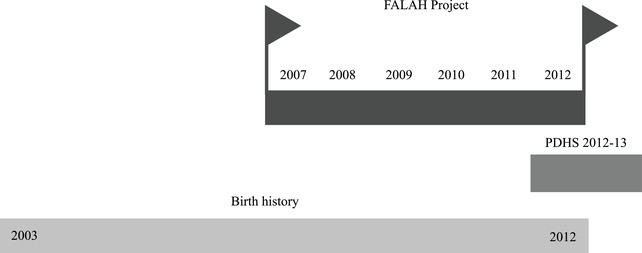
Analytical strategy NOTE: This figure shows the timeline of the FALAH project and the period for which we used the birth history data from the Pakistan Demographic and Health Survey 2012–2013. Although PDHS is a cross‐sectional survey, it collected information on birth histories of all the sampled women of reproductive age. This provided us an opportunity to compare interbirth intervals about five years before the FALAH project (2002–2007) and five years after the project was launched (2007–2012).

We limit the analysis to non‐first‐order births as the intervention was not targeting contraceptive use for first‐order births. We also restrict our sample to births that took place on or after 2003, thus covering births that occurred within five years of the start of the FALAH project (see Figure 2). In robustness checks, we show that the key findings are unaffected when we extend the sample to cover additional years.

## EMPIRICAL STRATEGY

Our first outcome of interest is the duration between successive births, measured in months. The second outcome is a binary measure of whether birth spacing was short as per WHO guidelines (i.e., less than 36 months (WHO 2005)). There is a lack of clarity in the literature on how short birth intervals should be defined. WHO recommends that women wait at least 24 months after the delivery of a live birth before conceiving another child (WHO 2005), implying that the gap between two births should be at least 33 months (= 24+9) (Pimentel et al. 2020). However, it is more convenient to collect information on the date of birth rather than the date of conception. Therefore, following Molitoris, Barclay, and Kolk (2019), we identify birth intervals shorter than 36 months as short birth intervals. (Note that our results do not change substantially even if a cutoff of 33 months is used).

To estimate the causal effect of the project on these outcomes, we compare the outcomes for multiple children born to the same mother before and after the project, using mother‐fixed effects. We estimate the coefficients in the following equation:

(1) $$Y_{ijt}=\alpha +\rho \;D_{ijt}+{X^{{}^{\prime }}}_{ijt}\beta +\lambda_{j}+\varepsilon_{ijt}$$

In this equation, *Y_ijt_* is the outcome for a child *i*, born to mother *j*, in year *t*. As mentioned above, we use interbirth interval both as a continuous variable and as a dichotomous variable. *D_ijt_* is a binary variable that measures exposure to the FALAH project and varies by births even for the same mother. ρ is the expected change in birth interval due to exposure to FALAH. *X_ijt_* represents characteristics of the child that can vary over time between multiple children born to the same mother. λ*_j_* are the mother‐fixed effects intended to capture confounding due to time‐invariant characteristics of the mother. ε*_ijt_* is the usual error term. We cluster the standard errors at the district level to allow arbitrary correlation between observations within a district.

D*_ijt_* warrants further discussion. As mentioned above, implementation of FALAH varied across districts in terms of roll‐in and duration (Capps et al. 2012; Jain et al. 2014). For each birth that took place between 2003 and 2012, we assign the birth to “exposed” or “not exposed” status (binary) based on the year of birth and the timing of the start of the project in the child's district of birth. We consider a birth exposed if the child was conceived while the project was being implemented in a particular district and not exposed otherwise (i.e., if the child was conceived prior to the date the project was rolled into the district). We use nine months prior to the child's date of birth as the date of conception.

As an illustration, take three children born to the same mother in Lasbella district, where the project started in June 2007 (Capps et al. 2012). Assume that two of those children were born before February 2008 (June 2007 plus nine months) and the third child was born after February 2008. The first two are not exposed to the program because the program had not started when they were conceived, whereas the third child is exposed. Note that we have information on *interbirth* interval only for the second and the third child; for the first child, we only know how much the mother waited between getting married and having the child. Our empirical strategy involves comparing the two intervals for the second child and the third child. In our main analysis, we classify all districts where FALAH was implemented at any point during the five‐year period (March 2008 and September 2012) as exposed, even if the project was not implemented for the entire period. We do so because the educational component of FALAH may have continued to influence persons in the project district even after its cessation. However, we also conduct a separate analysis by categorizing the districts as fully implemented and partially implemented.

Following previous studies using a similar empirical strategy (e.g., Østb*y* et al. 2018), the time‐varying factors that we control in our analysis include birth order, proportion of living sons (amongst living children), gender of the preceding birth, death of an older child, and age of the mother at the time of index child's birth. These covariates account for differences in birth spacing that can occur between births even for the same mother. Previous research has shown that birth spacing increases with birth order (Casterline and Odden 2016), as families approach their target number of children. Research also shows that as women get older, their birth intervals tend to get longer because of reduced fertility and higher chances of having achieved their desired family size (RamaRao, Townsend, and Askew 2006). Likewise, given a strong son preference in Pakistan (Zaidi and Morgan 2016; Winkvist and Akhtar 2000) and much of South Asia, spacing can change based on the number of sons a mother already has (Rahman and Davanzo 1993; Rossi and Rouanet 2015; Javed and Mughal 2020). Sex of the preceding birth can also impact duration to the next birth, with the birth of a son usually widening the duration (Jayachandran and Kuziemko 2011; Rossi and Rouanet 2015). The death of a child also reduces birth interval for the next child by truncating the temporary infertility that occurs when a woman is not menstruating and fully breastfeeding (a phenomenon called lactational amenorrhea) and because of parent's decision to replace the child who did not survive (Hossain, Phillips, and Legrand 2007; Bhalotra and van Soest 2008; van Soest and Rani Saha 2012).

The key assumption we need to make in order to interpret *ρ* as the estimate of the causal effect of FALAH is that, in absence of FALAH, a mother would have similar intervals between multiple births if the time‐varying factors mentioned above were to remain unchanged between births. We recognize that other features, such as the mother's education and household income, may change between births. Likewise, households may migrate from one region to another. Given the cross‐sectional nature of our data, it is not possible to control for these variables while retaining causal interpretation of the results. In a fixed‐effect model, these time‐invariant variables would drop. Therefore, we conduct heterogeneity analysis by mother's area of residence, education levels, and household wealth, to examine if the effect of the project varies along these dimensions—an important consideration for targeting policy interventions.

As discussed in the section Discussion and Conclusion, a key limitation of using mother fixed effects is that the estimated effect comes from a small subsample of mothers who had at least three children. An alternative approach to estimate the causal effect of FALAH would be to compare spacing in project and nonproject districts before and after the project, using a difference‐in‐differences (DID) framework. While health policy researchers have used this approach widely (Ryan, Burgess, and Dimick 2015), the approach seems inappropriate for this study. In Online Appendix B, we provide a detailed discussion of the tests we carried out and our rationale for choosing mother‐fixed effects over DID. Briefly, the parallel trends assumption required in DID is violated in the current study's context, likely because the districts where FALAH was rolled in first were also the districts with the shortest birth intervals, on average, before the project. In the section Discussion and Conclusion, we return to the issue of threats to the external validity of our findings from the use of mother fixed effects.

## RESULTS

### Main Results

Our main analytical sample consists of 18,414 non‐first‐order births contributed by 8,222 women (Table 1). (See Online Appendix Table A1 for how we derived this analytic sample.) The average interbirth interval is 31.5 months, and 70.7 percent of the birth intervals were short according to the WHO guidelines. The average child was a fourth child in their family. About half of the births were from the households that fell into the poorest two quintiles based on the wealth index (DHS calculates the wealth quintiles based on the distribution of the wealth index across the entire DHS sample). Two thirds of the births in the sample were given by mothers with no schooling, whereas only one in seven of the births was given by mothers with a secondary or higher level of education. About 41 percent of the births in the analytical sample took place in urban areas. Approximately 7.9 percent of the births (from 13.6 percent mothers) in the analytical sample were exposed to the FALAH project.

**TABLE 1:** Summary statistics for the analytic sample (*N* = 18,414)

Variable	Mean	SD
Preceding birth interval (months)	31.49	18.96
Birth spacing*		
Short interval	70.71	–
Nonshort interval	29.29	–
Exposure to FALAH intervention (%)	7.85	0.27
Birth order	4.34	2.25
Proportion of sons in the family (before focal birth)	0.49	0.36
Gender (boy = 1)	0.51	–
Child death (%)	9.25	0.29
Mother's age (at the time of birth)		
<25	29.16	–
25–30	32.74	–
30+	38.11	–
Mother's schooling (%)		
No schooling	65.90	–
Less than secondary	18.54	–
Secondary+	15.56	–
Quintiles of wealth index (%)		
Poorest	25.86	–
Poorer	21.29	–
Middle	19.57	–
Richer	17.35	–
Richest	15.94	–
Community‐level indicators (%)		
Urban residence	41.06	–

NOTE: Unit of analysis for this table is births

* Following the guidelines by WHO, interbirth intervals shorter than 36 months are considered as short birth intervals (WHO 2005).

In Table 2, we present results from estimating the coefficients in Equation (1), with interbirth interval (continuous) as the outcome, in a stepwise manner, adding a new covariate in each step, so that the reader can see the stability of the coefficient on FALAH (*ρ*). We start by controlling for the birth order, then added death of a child in the family (binary), proportion of sons among the older children born to the mother, sex of the preceding birth, and mother's age at the time of index birth. All columns include mother‐fixed effects.

**TABLE 2:** Mother‐fixed effect results for the effect of FALAH on interbirth intervals (continuous)

Variable	Model 1	Model 2	Model 3	Model 4	Model 5	Model 6	Model 7
FALAH	3.866***	3.308***	3.264***	3.267***	3.267***	2.453***	3.945***
	(0.904)	(0.876)	(0.858)	(0.858)	(0.857)	(0.754)	(0.782)
Birth order (two=0)							
Three		1.648***	1.606***	1.597***	1.632***	−0.857**	−0.920*
		(0.422)	(0.423)	(0.427)	(0.422)	(0.424)	(0.472)
Four		1.848***	1.814***	1.804***	1.851***	−3.502***	−3.285***
		(0.516)	(0.513)	(0.517)	(0.513)	(0.555)	(0.650)
Five		2.781***	2.755***	2.743***	2.783***	−5.297***	−5.082***
		(0.638)	(0.637)	(0.643)	(0.641)	(0.757)	(0.907)
Six+		1.932***	1.865**	1.850**	1.905***	−9.143***	−9.155***
		(0.722)	(0.724)	(0.726)	(0.716)	(0.957)	(1.140)
Child death			−2.566***	−2.542***	−2.626***	−2.429***	−2.120***
			(0.419)	(0.439)	(0.435)	(0.429)	(0.472)
Proportion of sons				0.233	−0.559	−0.211	−0.331
				(0.705)	(0.857)	(0.841)	(1.009)
Sex of preceding birth (girl = 0)					0.458	0.283	0.358
					(0.354)	(0.342)	(0.398)
Mother's age (<25 years)							
25–30						8.585***	8.648***
						(0.602)	(0.684)
30+						16.333***	16.831***
						(1.116)	(1.245)
Constant	31.18***	29.737***	30.00***	29.89***	30.02***	26.25***	25.71***
	(0.071)	(0.383)	(0.379)	(0.481)	(0.493)	(0.573)	(0.648)
*R*‐squared (overall)	0.0002	0.004	0.010	0.011	0.010	0.094	0.094
*R*‐squared (within)	0.004	0.008	0.010	0.010	0.010	0.052	0.056
*F* statistic	18.29	8.24***	10.72***	9.78***	8.91***	28.23***	28.77***
Number of births	18,414	18,414	18,414	18,414	18,414	18,414	14,141

NOTES: ^*^ *p* < 0.10, ^**^ *p* < 0.05, ^***^ *p* < 0.01. This table shows coefficients and standard errors from a regression of birth interval on the variables shown. All models include mother‐fixed effects. Standard errors are clustered at the district level (*n* = 121). In Models 1–6, the districts where FALAH was implemented either fully (i.e., for all five years) or partially are considered exposed to FALAH. In Model 7, only the districts where FALAH was implemented for all five years are considered exposed. To illustrate the interpretation of the coefficients reported in the table, in Model 6, exposure to FALAH increased birth intervals by 2.5 months.

For interpretation, we focus on the results from the fully specified regression (Model 6). Based on that regression, exposure to the FALAH project resulted in an increase in interbirth intervals by 2.4 months after controlling for the time‐variant factors. Substantively, this is a large effect, which translates to an increase in birth intervals of approximately 9.3 percent from the mean (=100 × 2.45/26.25).

The coefficients on the covariates are in the expected direction except in the case of the regression that includes both birth order and mother's age. For example, the death of a child in the family is negatively associated with birth interval; on average, the occurrence of the death of a child in the family reduces the birth interval before a successive birth by 2.4 months. We find no association between the proportions of sons a mother already has and the birth interval before a subsequent birth. Having a son is also not associated with an increase in the next birth interval. In the regression that does not include mother's age (Model 5), birth order is positively associated with birth interval. However, this relationship is reversed once we control for mother's age, which is strongly and positively associated with birth interval (Model 6). While the change in the association between birth order and birth interval after we control for mother's age is surprising, it may be due to the high correlation between birth order and mother's age (*r* = 0.63).

The main findings do not change when we examine the effect on short intervals (the binary measure). After controlling for potential time‐varying confounders, we find that exposure to FALAH reduced the proportion of short intervals by 7.1 percentage points (Table 3). Given that 70.7 percent of births in the sample are short, the change represents an effect of about 8 percent (=100 × 7.1/85.8). Here, too, the coefficients on the covariates are in the expected direction except on birth order when mother's age is included in the regression.

**TABLE 3:** Mother‐fixed effect results for the effect of FALAH on short birth intervals (binary)

	Model 1	Model 2	Model 3	Model 4	Model 5	Model 6	Model 7
FALAH	−0.111***	−0.091***	−0.091***	−0.091***	−0.091***	−0.071***	−0.139***
	(0.030)	(0.029)	(0.028)	(0.028)	(0.028)	(0.026)	(0.026)
Birth order (two=0)							
Three		−0.065***	−0.064***	−0.063***	−0.064***	−0.006	−0.001
		(0.011)	(0.011)	(0.011)	(0.011)	(0.011)	(0.011)
Four		−0.066***	−0.066***	−0.064***	−0.066***	0.059***	0.047***
		(0.015)	(0.015)	(0.015)	(0.015)	(0.015)	(0.016)
Five		−0.091***	−0.090***	−0.089***	−0.090***	0.100***	0.082***
		(0.018)	(0.018)	(0.018)	(0.018)	(0.019)	(0.022)
Six+		−0.072***	−0.070***	−0.069***	−0.071***	0.192***	0.186***
		(0.020)	(0.020)	(0.020)	(0.020)	(0.022)	(0.026)
Child death			0.053***	0.050***	0.054***	0.049***	0.040***
			(0.013)	(0.014)	(0.014)	(0.014)	(0.015)
Proportion of sons				−0.027	0.006	−0.003	−0.003
				(0.022)	(0.027)	(0.026)	(0.029)
Sex of preceding birth (girl = 0)					−0.019*	−0.015	−0.018
					(0.011)	(0.010)	(0.012)
Mother's age (<25 years)							
25–30						−0.196***	−0.193***
						(0.015)	(0.017)
30+						−0.390***	−0.395***
						(0.028)	(0.031)
Constant	0.716***	0.769***	0.763***	0.775***	0.770***	0.858***	0.874***
	(0.002)	(0.011)	(0.010)	(0.013)	(0.013)	(0.015)	(0.017)
*R*‐squared (overall)	0.0001	0.004	0.008	0.009	0.008	0.063	0.065
*R*‐squared (within)	0.0042	0.009	0.010	0.011	0.011	0.039	0.044
*F* statistic	14.07	9.75***	9.07***	8.98***	7.99***	24.99***	25.04***
Number of births	18,414	18,414	18,414	18,414	18,414	18,414	14,141

NOTES: ^*^ *p* < 0.10, ^**^ *p* < 0.05, ^***^ *p* < 0.01. This table shows coefficients and standard errors from a regression of a binary indicator of interbirth intervals on the variables shown. All models include mother‐fixed effects. Standard errors are clustered at the district level (*n* = 121)). In Models 1–6, the districts where FALAH was implemented either fully (i.e., for all five years) or partially are considered exposed to FALAH. In Model 7, only the districts where FALAH was implemented for all five years are considered exposed. To illustrate the interpretation of the coefficients reported in the table, in Model 6, exposure to FALAH decreased the likelihood of a mother practicing short birth interval by 7.1 percentage points.

### Heterogeneous Effects by Duration of Implementation

Recall that, in our main analysis, all districts where the project was implemented count as such, irrespective of the duration of the implementation. For a project of this nature, it is natural to expect some variation in the quality of implementation—especially when the duration of the implementation differs. To see if the magnitudes of the effects differ by exposure to this duration, we conduct a separate analysis by counting as project districts only the districts where FALAH was implemented for all five years. The nonproject districts are districts where the project was never implemented. Districts where the project was implemented for fewer than five years were eliminated from the analysis.

Results from estimating equation (1) using this new classification of districts are in column 7 of Tables 2 and 3 (and in Online Appendix Figure A1 for direct comparison across the types of districts: non‐FALAH, partial implementers, and full implementers). As expected, the effect of the project on districts where it was implemented for all five years is higher—by approximately 1.5 months—than in districts where the project was implemented either partially or fully. Similarly, in the case of the binary outcome variable, the mothers practicing short birth interval decreased by 13.9 percentage points where FALAH project was implemented for the entire duration of five years compared to 7.1 percentage points when we also included districts where FALAH was implemented partially. Online Appendix Figure A1 shows that, at the 5 percent significance level, there was a statistical difference in interbirth interval between non‐FALAH districts and full implementers, but no such difference between other pairs of districts. In terms of proportion of short intervals, however, effects differed statistically both between non‐FALAH and full implementers and between full implementers and partial implementers.

### Heterogeneous Effects by Geography, Education, Age, and Wealth

In Table 4, we show the estimates of the effect of FALAH, separately for the continuous and binary measures, by women's urban versus rural status and education levels at the time of the survey. The project had a higher impact in rural areas than in urban areas (Panel A). In rural areas, FALAH raised the average interbirth interval by approximately 3.2 months, which translates to an increase of 10.6 percent at the mean. Similarly, the project reduced the proportion of short intervals in rural areas by 8.5 percentage points. The project had no effect in urban areas.

**TABLE 4:** Heterogeneous effects of FALAH, by women's geographic area and schooling

Independent variables →	Interbirth interval, months (continuous)		Interbirth interval short (binary)	
Panel A: Geographic area				
	Urban	Rural	Urban	Rural
FALAH	0.972	3.219***	−0.050	−0.085***
	(0.866)	(1.100)	(0.036)	(0.032)
Overall mean	33.05	30.40	0.67	0.73
Percent effect at the mean	2.94	10.59	−7.46	−11.64
*N*	7,560	10,854	7,560	10,854

Panel B: Mother's schooling						
	No schooling	Less than secondary	Secondary+	No schooling	Less than secondary	Secondary+
FALAH	2.580***	0.603	6.873***	−0.077***	0.009	−0.228***
	(0.826)	(1.551)	(2.011)	(0.029)	(0.047)	(0.078)
Overall mean	30.71	32.09	34.03	0.72	0.69	0.65
Percent effect at the mean	8.4	1.88	20.20	−10.69	1.30	−35.08
*N*	12,134	3,414	2,866	12,134	3,414	2,866

NOTES: ^*^ *p* < 0.10, ^**^ *p* < 0.05, ^***^ *p* < 0.01.

This table shows the heterogeneous effect of FALAH by geographic area, mother's schooling, and mother's age, separately for the interbirth interval (continuous) and short interval (binary). Each coefficient is from a separate regression. The following covariates are included in the models but not shown in the table: birth order, mother's age (at the time of birth), child death, proportion of sons, and sex of preceding birth. All models include mother‐fixed effects. Standard errors are clustered at the district level (*n* = 121). Using the coefficients and the overall mean of the outcome measure for the respective group, we calculate the size of the effect for that group. For example, FALAH reduced interbirth intervals among urban women by 2.9 percent (=100 × 0.97/33.05). Likewise, it reduced the proportion of short birth intervals by 7.5 percent.

The project had the largest effect among women who had secondary or higher levels of education, followed by those who had no schooling (Panel B). This was true for both outcome measures. At the respective means, FALAH increased interbirth interval by 20.2 and 8.4 percent for women with secondary or higher levels of education and those with no education, respectively. The corresponding effects on the proportion of short intervals were 35.1 and 10.7 percent, respectively. The project had no effect on the interbirth interval or the proportion of short intervals for women with a primary or lower secondary level of education.

**FIGURE 3:**
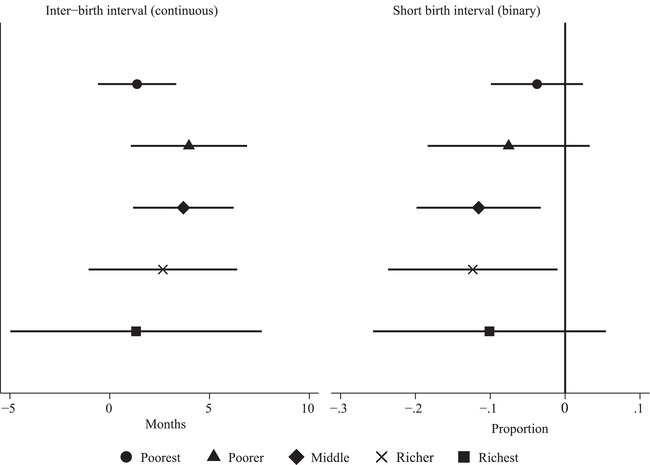
Heterogeneous effects of FALAH, by household wealth index NOTES: The figure shows the effect of exposure to FALAH on interbirth intervals (in months) and the proportion of short birth intervals, by the quintiles of the wealth index. The regression results underlying these figures are in Online Appendix Table A2.

In terms of the wealth quintiles, the project had the most pronounced effects on the interbirth interval for women in the middle of the wealth distribution (Figure 3). Based on the regression results underlying Figure 3 (Online Appendix Table A2), for women belonging to households in quintiles 2 (poorer) and 3 (middle), FALAH increased interbirth intervals by 4.0 and 3.7 months, respectively. These effects are equal to 12–13 percent of the effect at the mean (e.g., for those in quintile 2, the effect is 100 × 4.0/30.8 = 12.9 percent). There is no impact of the intervention on the inter‐birth intervals of the women in the poorest (quintile 1) and the top two quintiles (richer and richest). The lack of an effect among women in the poorest quintile does not seem to be due to a small sample size as the standard error on the coefficient is smaller than in other quintiles. We are, however, less certain about the lack of an effect on the richest quintile, as the coefficient is less precise. In terms of the reductions in the proportion of short birth intervals, FALAH's effect was the most pronounced among mothers in the middle wealth quintile.

### Robustness Check

We checked the robustness of our main results in a number of ways. First, using the previous rounds of the Pakistan Demographic and Health Survey data (PDHS 2006–2007), we conducted regressions similar to those reported in Tables 2 and 3, but by assuming that the program was implemented in 2004 in the same set of districts in which FALAH was subsequently implemented. We controlled for the same set of time‐varying covariates and used mother‐fixed effects. This falsification test confirms (see Online Appendix Table A3) that the positive effects we found in the main analyses are unlikely to have occurred by chance.

Recall that in the main analysis, we restrict the analysis to births that occurred within five years of the start date of FALAH. As a second robustness check, we extended this duration to six years prior to FALAH. Using this expanded analytical sample of birth histories from 2002 to 2012, we were still able to find a significant positive effect of the FALAH project on both measures of the outcome (Online Appendix Table A4 panel A).

As discussed in the section Empirical Strategy, the estimated effect in our analysis is based on the subsample of women who were exposed to FALAH (the “switchers”). We conducted further analysis by restricting the sample to women in the program districts, thus removing any noise from the nonswitchers. The results from these analyses, presented in Online Appendix Table A4 Panel B, are not substantively different from the main results.

## DISCUSSION AND CONCLUSION

In this paper, we exposed a large family planning intervention in Pakistan, called FALAH, to a rigorous evaluation technique and assessed its effect on birth spacing. Deviating from previous family planning programs in Pakistan, the FALAH project emphasized birth spacing, as opposed to limiting family size, as the primary purpose of contraceptive use. Our findings suggest that this repositioning of the goal of contraceptive use resonated well with Pakistani couples. Although some of the estimated effects may be due to other aspects of the project, such as supply‐side strengthening, given the focus on messaging, it is reasonable to conjecture that much of the estimated effect is due to the nature of the messaging. These findings are important for the context of Pakistan where fertility rates have been stubbornly high, contraceptive uptake has been low, and birth intervals have been narrow, all of which adversely affect maternal and child health.

Our results agree with previous evaluations, although those evaluations have focused on different outcomes, and it is difficult to interpret their findings as causal. Using data collected during baseline and endline surveys of the married women of reproductive age, Mahmood (2012) shows that contraceptive prevalence increased from 29.4 percent in 2008–2009 to 37.9 percent in 2011–2012 in selected FALAH districts. Using the same data, Jain et al. (2014) show that the unmet need for spacing decreased from 15 percent in 2008–2009 to 11 percent in 2011–2012. Ashfaq and Sadiq (2015) assess the impact of FALAH activities specifically targeting men. They show that men who were exposed to the activities, such as the community‐level male group meetings, sermons by sensitized mosque leaders, interactive community theatre, and an electronic media campaign, experienced a positive impact on several family planning‐related attitudes and behaviors, including husbands’ willingness to discuss contraception with their wives, and their approval of family planning and contraceptive use. Another analysis by Mir and Shaikh (2013) shows that 38 percent of healthcare providers trained under the FALAH project accurately understood the birth spacing concept as per the WHO recommendation of HTSP compared to only 5 percent of the untrained providers. The qualitative component of Mir and Shaikh (2013) confirms that the training provided by the FALAH project helped change individuals’ perceptions about the permissibility of family planning in Islam. While all of these studies have significant limitations—primarily that we cannot fully attribute the estimated changes in outcomes to FALAH—the findings imply that it is reasonable to expect positive effects of FALAH on birth spacing—as we find in this study.

Although we overcome the major limitations of previous studies, our study also has a number of limitations. The most important limitation relates to our use of mother fixed effects, which improve internal validity at the expense of generalizability (Hutcheon and Harper 2019; Miller, Shenhav, and Grosz 2019). There is also no guarantee that control for confounding is complete (Hutcheon and Harper 2019). In the context of our study, the estimated effects come from a subsample of women who have at least three children, with at least one of them conceived before FALAH and another conceived after FALAH—the so‐called switchers in the econometric literature. The TFR in Pakistan is 3.6 births per woman, which makes the analysis more appropriate here than in many other settings with a lower TFR. Nonetheless, the switchers may differ from the overall nationally representative sample in terms of the broad determinants of birth spacing, such as education and household wealth, thus significantly limiting the finding's external validity. Indeed, a comparison of women who were exposed to FALAH and those who were not reveals that the two groups are different from each other in many demographic characteristics (Online Appendix Table A5). On average, women who were exposed to FALAH are younger, more rural, less educated, and poorer. Their birth intervals are also shorter. If the women who were exposed to the project are less receptive to health messages provided as part of the project (given their characteristics), our estimates are downward biased. Conversely, if the scope for improving birth intervals is higher for these women—given that their current intervals are relatively shorter—our estimates are upward biased. Overall, it is difficult to ascertain the net bias from the differences in characteristics of women exposed to FALAH and those not exposed to it. Irrespective of the direction of the bias, it is worth reiterating that the project's effect on the overall population of women in Pakistan can be potentially very different from what we have documented.

Furthermore, our findings should be interpreted as intent‐to‐treat effects (rather than treatment‐on‐the‐treated effects), as a woman's exposure to FALAH is defined by her district of residence rather than by whether she was a direct beneficiary of the project.

Second, due to the data limitations, we could not estimate the effect of FALAH on contraceptive use. Even though FALAH focused on birth spacing messages, these messages were about the use of contraceptives in order to achieve longer birth intervals. PDHS 2012–2013 contains information on contraceptive use between 2007 and 2012–2013 (i.e., during the five years preceding the survey). Therefore, we only have contraceptive use data *after* FALAH was implemented in many districts.

A third limitation relates to censoring. Consider a woman who was exposed to FALAH and, as a result, decided to either delay the next birth for more than five years or stop childbearing. Our analysis is set up to compare the birth intervals for births that occurred (from the same mother) pre‐ and post‐FALAH implementation. As such, it does not account for women who did not have a birth during the post‐FALAH period. This implies that our estimates may be biased downward because of censoring.

Fourth, birth history data obtained through retrospective reporting are prone to multiple biases (Rustein and Rojas 2006; Moultrie, Sayi, and Timæus 2012) especially in a country like Pakistan where vital statistics systems are weak. The biases introduced by retrospective birth history could affect our estimates, although the direction of the bias is unclear.

Finally, we are unable to account for abortion, unintentional pregnancy loss, or stillbirths in our analysis. The prevalence of abortions or pregnancy loss in a population can lead to an overestimation of the length of birth intervals (Rossi and Rouanet 2015). Similarly, if the sex‐selected abortions are common, the birth intervals could be longer. Even though studies have found no evidence of sex‐selective abortions in Pakistan (Zaidi and Morgan 2016), Sathar et al. (2014) show that nearly half of all pregnancies in Pakistan are unintended and more than half of these end in abortion. Our findings will be unaffected by abortion if abortion does not take place between the two births we compare for the same mother or if a prior abortion (i.e., one that may have taken place before the first child) does not affect decisions about subsequent spacing. Nonetheless, abortion is an important consideration future research should address.

In addition to addressing the limitations above, future studies can fill the gap in two other important areas. Immediate among these areas is an exploration of the mechanisms behind the success of the FALAH project, perhaps through a deeper qualitative study to analyze what aspects of the project were successful in promoting birth spacing. FALAH also had a few supply‐side components, such as improving the availability of contraceptives and training providers for better management of side effects, although behavioral change communication was a defining feature of the program as discussed earlier. It is reasonable to expect that some of the effect we estimate may have come from these other components.

Second, and more generally, a comparative study of messaging in family planning programs in Pakistan and other high‐fertility settings that provides broad as well as context‐specific lessons for policymakers seems critical for the design of future family planning programs. This line of research can build on the Pakistan‐based studies referenced above as well as evidence from other countries where policymakers have attempted to reframe goals of family planning interventions to fit their contexts. For instance, a qualitative study evaluating the success of the family planning program in Rwanda shows that one of the reasons for the success of the family planning program there is that the government promoted family planning program as a tool to empower individuals and families rather than as a program with a motive to curb population growth (Schwandt et al. 2018). Other studies also acknowledge that the recent success of family planning programs in Ethiopia, Kenya, Malawi, Rwanda, Tanzania, and Nepal can be attributed to the messaging of the family planning programs that promoted contraception largely as a child and maternal health intervention rather than a program intended to curb population growth (AFIDEP 2013; Olson and Piller 2013; Brunson 2020). For effective policy design, these family planning programs need to be evaluated rigorously.

Finally, the need for implementing large‐scale projects of this nature in a way that allows for rigorous ex post evaluation cannot be overstated. Many of the limitations of our study relate to the nonrandom roll‐in of the FALAH project. In the future, implementers should consider other approaches, such as rolling in the projects randomly rather than into more problematic areas first, so that alternative evaluation methods which allow for greater generalizability of results can be used.

## Supporting information

Online Appendices
