# Supplementary material for: The Effect of Reframing the Goals of Family Planning Programs from Limiting Fertility to Birth Spacing: Evidence from Pakistan
Source: Stud Fam Plann. 2021 May 20;52(2):125–42. doi: 10.1111/sifp.12155 (PMC8362150; doi:10.1111/sifp.12155)
Supplement: Supplementary file 1 — Online Appendices [file SIFP-52-125-s001.docx]

**APPENDICES**

**Appendix A**

**Figure A1. Comparison of effects across districts based on the duration of implementation**

1. **Inter-birth interval**

1. **Short birth interval (binary)**

Notes: These figures show the average months of inter-birth interval and the probability of short birth interval by the duration of exposure to FALAH. The numbers underlying these figures were obtained from estimating equation (1), where exposure is a categorical variable (no exposure, partial exposure, and exposure for all five years). 95% confidence intervals are shown.

**TABLE A1 Derivation of the analytic sample for the main analysis**

|  | **N** |
| --- | --- |
| Total number of births delivered by the interviewed mothers in PDHS 2012-13 | 50,238 |
| Number of births after randomly selecting one of the twin/triplet births | 49,720 |
| Number of births between 2003 and 2012 | 23,513 |
| Non-first-order births (as outcome of interest is preceding birth interval) | 18,414 |
|  |  |
| Total number of mothers in the analytical sample | 8,222 |
|  |  |
| Source: Pakistan Demographic and Health Survey 2012-13 |  |

**TABLE A2 Mother-fixed effect results for the effect of FALAH, by wealth quintile of the household**

|  | **Quintiles of wealth index** | | | | |
| --- | --- | --- | --- | --- | --- |
|  | **Poorest** | **Poorer** | **Middle** | **Richer** | **Richest** |
|  | | | | | |
| **Panel A: Inter-birth interval (continuous)** | | | | | |
|  | 1.369 | 3.967*** | 3.690*** | 2.663 | 1.319 |
| FALAH | (0.990) | (1.470) | (1.271) | (1.877) | (3.170) |
|  |  |  |  |  |  |
| Constant | 26.597*** | 27.268*** | 24.758*** | 26.716*** | 24.185*** |
|  | (0.935) | (1.222) | (1.167) | (1.484) | (1.445) |
|  |  |  |  |  |  |
| Overall mean | 29.29 | 30.79 | 30.73 | 32.43 | 35.87 |
|  |  |  |  |  |  |
| % effect at the mean | 4.67 | 12.88 | 12.01 | 8.21 | 3.68 |
|  | | | | | |
| **Panel B: Short birth intervals (binary)** | | | | | |
|  |  |  |  |  |  |
| FALAH | -0.038 | -0.075 | -0.116*** | -0.123** | -0.101 |
|  | (0.031) | (0.055) | (0.042) | (0.057) | (0.078) |
|  |  |  |  |  |  |
| Constant | 0.860*** | 0.833*** | 0.871*** | 0.828*** | 0.935*** |
|  | (0.026) | (0.033) | (0.034) | (0.041) | (0.043) |
|  |  |  |  |  |  |
| Overall mean | 0.76 | 0.72 | 0.72 | 0.68 | 0.62 |
|  |  |  |  |  |  |
| % effect at the mean | -5.00 | -10.42 | -16.11 | -18.09 | -16.29 |
|  |  |  |  |  |  |
| N | 4,761 | 3,920 | 3,603 | 3,194 | 2,936 |

Notes: * p<0.10, ** p<0.05, *** p<0.01.

This table shows heterogeneous effect of FALAH by wealth quintile of the household separately for inter-birth interval (continuous) and short interval (binary). Each coefficient is from a separate regression. The following covariates are included in the models but not shown in the table: birth order, mother’s age (at the time of birth), child death, proportion of sons, and sex of preceding birth. All models include mother-fixed effects. Standard errors are clustered at the district level (n=121).

**TABLE A3 Falsification Test – Mother-fixed effects results for the effect of FALAH assuming FALAH was implemented in 2004**

|  | **Model 1** | **Model 2** | **Model 3** | **Model 4** | **Model 5** | **Model 6** |
| --- | --- | --- | --- | --- | --- | --- |
| **Panel A: Inter-birth interval (continuous)** | | | | | | |
|  |  |  |  |  |  |  |
| FALAH* | -3.768*** | -3.235*** | -3.156*** | -3.153*** | -3.150*** | -2.526*** |
| Birth order (two=0) |  |  |  |  |  |  |
| Three |  | 1.775*** | 1.731*** | 1.726*** | 1.761*** | -0.784* |
| Four |  | 2.076*** | 2.041*** | 2.035*** | 2.081*** | -3.380*** |
| Five |  | 3.182*** | 3.152*** | 3.145*** | 3.185*** | -5.071*** |
| Six+ |  | 2.496*** | 2.424*** | 2.416*** | 2.470*** | -8.825*** |
| Child death |  |  | -2.573*** | -2.558*** | -2.641*** | -2.437*** |
| Proportion of sons |  |  |  | 0.150 | -0.631 | -0.263 |
| Sex of preceding birth (girl=0) |  |  |  |  | 0.452 | 0.276 |
| Mother’s age  (<25 years) |  |  |  |  |  |  |
| 25-30 |  |  |  |  |  | 8.649*** |
| 30+ |  |  |  |  |  | 16.468*** |
| Constant | 31.621*** | 29.843*** | 30.115*** | 30.045*** | 30.172*** | 26.344*** |
|  |  |  |  |  |  |  |
| **Panel B: Short birth intervals (binary)** | | | | | | |
|  |  |  |  |  |  |  |
| FALAH* | 0.137*** | 0.119*** | 0.117*** | 0.117*** | 0.117*** | 0.101*** |
| Birth order (two=0) |  |  |  |  |  |  |
| Three |  | -0.067*** | -0.066*** | -0.066*** | -0.067*** | -0.007 |
| Four |  | -0.071*** | -0.070*** | -0.070*** | -0.071*** | 0.057*** |
| Five |  | -0.100*** | -0.100*** | -0.098*** | -0.100*** | 0.095*** |
| Six+ |  | -0.085*** | -0.083*** | -0.082*** | -0.084*** | 0.185*** |
| Child death |  |  | 0.053*** | 0.051*** | 0.054*** | 0.049*** |
| Proportion of sons |  |  |  | -0.024 | 0.008 | -0.001 |
| Sex of preceding birth (girl=0) |  |  |  |  | -0.019* | -0.014 |
| Mother’s age  (<25 years) |  |  |  |  |  |  |
| 25-30 |  |  |  |  |  | -0.197*** |
| 30+ |  |  |  |  |  | -0.393*** |
| Constant | 0.702*** | 0.763*** | 0.757*** | 0.769*** | 0.764*** | 0.853*** |
| N | 18,414 | 18,414 | 18,414 | 18,414 | 18,414 | 18,414 |

Notes: * p<0.10, ** p<0.05, *** p<0.01. This table shows coefficients from a regression of outcomes on exposure to FALAH*, where FALAH* is assumed to go into effect in March 2004. All models include mother-fixed effects. Standard errors are clustered at the district level (n=121).

**TABLE A4 Robustness Check – Mother-fixed effect results for the effect of FALAH intervention on birth intervals**

| **Independent variables →** | **Inter-birth interval**  **(continuous)** | | **Short interval**  **(binary)** | |
| --- | --- | --- | --- | --- |
|  | **Bivariate** | **Multivariate** | **Bivariate** | **Multivariate** |
| Panel A: Robustness check **–** Additional years included | | | | |
| FALAH | 4.572*** | 2.902*** | -0.121*** | -0.074*** |
|  | (1.063) | (0.886) | (0.033) | (0.028) |
|  |  |  |  |  |
| Constant | 31.110*** | 25.777*** | 0.718*** | 0.865*** |
|  | (0.075) | (0.596) | (0.002) | (0.015) |
|  |  |  |  |  |
| R-squared (within) | 0.0051 | 0.056 | 0.0044 | 0.039 |
| R-squared (overall) | 0.0002 | 0.093 | 0.0001 | 0.061 |
| F statistic | 18.50 | 37.79*** | 13.69 | 38.34*** |
| N | 20,401 | 20,401 | 20,401 | 20,401 |
| Panel B: Robustness check **–** Sample restricted to the program districts only | | | | |
| FALAH | 3.866*** | 3.330*** | -0.111*** | -0.094*** |
|  | (0.913) | (0.854) | (0.030) | (0.028) |
|  |  |  |  |  |
| Constant | 30.874*** | 27.926*** | 0.732*** | 0.830*** |
|  | (0.225) | (0.985) | (0.007) | (0.027) |
|  |  |  |  |  |
| R-squared (within) | 0.0141 | 0.047 | 0.0132 | 0.041 |
| R-squared (overall) | 0.0002 | 0.082 | 0.0003 | 0.057 |
| F statistic | 17.91 | 9.08*** | 13.78 | 8.99*** |
| N | 5,866 | 5,866 | 5,866 | 5,866 |

Notes: * p<0.10, ** p<0.05, *** p<0.01.

This table shows coefficients and standard errors from a regression of birth intervals on FALAH. All models include mother-fixed effects. Standard errors are clustered at the district level (n=121). The following covariates are included in the multivariate models but not shown in the table: birth order, mother’s age (at the time of birth), child death, proportion of sons, and sex of preceding birth. In Panel A, the regressions were estimated on a sample of births that took place between six years prior and five years after the program initiation. This is meant as a robustness check. Recall that in the main analysis, we limit the sample to births that took place within five years of the program initiation. In Panel B, we restrict the sample to the women living in program districts only, thus removing any noise in the estimates from women in non-program districts (the so-called ‘non-switchers’).

**TABLE A5 Background characteristics of mothers, by exposure to FALAH (N=8,222)**

| Variables | Switchers^*^ | Non-switchers |
| --- | --- | --- |
| Number of mothers | 1,124 | 7,098 |
| Preceding birth interval (months) | 32.20 | 35.48 |
| Birth spacing (%) |  |  |
| Short interval | 72.95 | 65.75 |
| Non-short interval | 27.05 | 34.25 |
| Mother's age (at the time of the survey) | 30.91 | 33.20 |
| Number of children (at the time of the survey) | 4.20 | 4.18 |
| Urban residence (%) | 34.34 | 45.78 |
| Mother's schooling |  |  |
| No schooling | 71.26 | 58.32 |
| Less than secondary | 16.37 | 20.34 |
| Secondary+ | 12.37 | 21.34 |
| Quintiles of wealth index |  |  |
| Poorest | 32.74 | 19.84 |
| Poorer | 21.35 | 20.03 |
| Middle | 19.04 | 19.58 |
| Richer | 16.73 | 18.95 |
| Richest | 10.14 | 21.61 |
| Province/territory |  |  |
| Balochistan | 13.35 | 29.33 |
| Khyber Pakhtunkhwa | 30.43 | 18.47 |
| Punjab | 28.11 | 19.22 |
| Sindh | 28.11 | 13.83 |
| Gilgit-Baltistan | 0 | 11.76 |
| Islamabad | 0 | 7.38 |

Notes: This table is prepared based on the analytical sample. Unit of analysis for this table is mothers. Mothers who had at least one non-first-order birth with exposure to FALAH and at least one non-first-order birth within five years before FALAH started is categorized as a Switcher for our analysis.

**Appendix B**

As mentioned in the main text, health policy researchers have used difference-in-differences (DID) approach widely (Ryan et al. 2015). In the current study, the method would involve comparing the change in birth intervals in FALAH and non-FALAH districts before and after FALAH. The key identifying assumptions of DID are “common shocks” and “parallel trends” (Angrist and Pischke 2008; Ryan et al. 2019). In the study’s context, the “common shocks” assumption would be that spacing in project and non-project districts will not be affected differentially by phenomena (e.g., policy changes at the national level) other than by FALAH itself. The parallel trends assumption holds that, although the spacing may differ between project and non-project districts before it was implemented, their trends would be the same. If these assumptions hold, spacing in project and non-project districts would change at the same rate after the project is implemented. Our understanding of the project and a formal assessment using the data from the 2006 PDHS—collected before FALAH was implemented—suggests that these assumptions are unlikely to hold.

The selection of districts was non-random. In fact, the selection of districts was based on lower contraceptive prevalence rate and high unmet need, therefore, the project districts were predominantly rural districts (Capps *et al*, 2012). Not surprisingly, the characteristics of women in project and non-project districts differed significantly before the program (Appendix Table B1). Given the sharp differences in these characteristics alone, it is unlikely that spacing in project and non-project districts would change at the same rate after FALAH.

To test the parallel trends assumption, we compare spacing in FALAH and non-FALAH districts between 2003 and 2007, after controlling for a wide range of covariates related to the mother and the birth. The results shows that the pre-program trends were different even before the program was introduced. This was true for the continuous measure of inter-birth interval (**Appendix Figure B1**) as well as its binary measure (**Appendix Figure B2**).When the pre-program trends are not parallel, one way to model DID is to allow differential linear trends between program and non-program districts by specifying time trends using flexible polynomials or to use matching estimators. Ryan *et al.* (2019) provide details on these approaches and note that these approaches, too, have weaknesses. For example, specifying time trends using flexible polynomials may attenuate the post-program slope for districts with a stronger pre-program trend (in our case, project districts). More importantly, Ryan *et al.* (2019) assess the performance of various approaches to addressing non-parallel trends. They find that DID with matching performs the best in the two measures they assessed. In our study’s context, matching would involve finding individuals from project and non-project districts who are similar to each other on a set of covariates and comparing their spacing before and after FALAH. Note that using mother fixed effects—where we compare the same individual before and after FALAH—is a refined version of matching.

Overall, while DID would have allowed us to utilize the between-district variation in exposure to the project, the assumptions required for causality are much weaker when using mother fixed effects. As discussed in the main text, with mother fixed effects, the estimates of the effect are derived from a small subset of “switchers”—mothers whose spacing changed before and after the program. Nonetheless, by comparing switchers to the entire sample, we are able to comment on the external validity of the findings.

**References for Appendix B**

Angrist, J. D. and Pischke, J. S. (2008) *Mostly harmless econometrics: An empiricist’s companion*, *Mostly Harmless Econometrics: An Empiricist’s Companion*. Princeton University Press. doi: 10.1111/j.1475-4932.2011.00742.x.

Capps, J. M. *et al.* (2012) *Family Advancement For Life and Health (FALAH) Evaluation Report*. Islamabad, Pakistan. Available at: https://files.globalwaters.org/water-links-files/Family Advancement For Life and Health (FALAH) Evaluation Report.pdf.

Ryan, A. M. *et al.* (2019) ‘Now trending: Coping with non-parallel trends in difference-in-differences analysis’, *Statistical Methods in Medical Research*, 28(12), pp. 3697–3711. doi: 10.1177/0962280218814570.

**Figure B1. Trends in inter-birth intervals (continuous) in FALAH and non-FALAH districts before 2007**

Notes: This figure shows that trend in inter-birth interval in FALAH and non-FALAH districts before the project was rolled in. The results presented in this figure are margins-plot obtained after estimating the following regression equation:

$$Y_{i}=\beta_{0}+\beta_{1}\left( {FALAH}_{i}\times{Birthyear}_{i} \right)+\beta_{n}X_{i}+\varepsilon_{i}$$

Y_i_ is inter-birth interval for birth *i,* $\beta_{1}$ is the coefficient of the interaction between FALAH district status and year of birth. $\beta_{n}$ is the coefficient vector of the following covariates: urban residence, wealth quintile of the household, mother’s education, father’s education, birth order, mother’s age, death of any child in the family, proportion of sons in the family, sex of preceding birth. The districts where FALAH was partially implemented are not included.

**Figure B2. Trends in binary measure of spacing in FALAH and Non-FALAH districts before 2007**

Notes: See notes to Figure B1 above. The only difference between this figure and Figure B2 is that the outcome in this case is binary (=1 if birth interval is less than 36 months).

**Table B1. Comparison of key demographic variables between FALAH and Non-FALAH districts before implementation, PDHS 2006-07**

| Variables | FALAH districts | Non-FALAH districts | p-value |
| --- | --- | --- | --- |
| Number of mothers | 1,074 | 5,533 |  |
| Mother's age (at the time of survey) | 32.4 | 33.5 | <0.001 |
| Number of children (at the time of survey) | 4.1 | 3.9 | <0.001 |
| Mother's schooling |  |  |  |
| No schooling | 76.7 | 66.1 | <0.001 |
| Less than secondary | 16.9 | 21.1 |  |
| Secondary+ | 8.5 | 12.8 |  |
| Father’s schooling |  |  |  |
| No schooling | 40.9 | 35.5 | <0.001 |
| Primary | 21.7 | 15.9 |  |
| Secondary | 20.5 | 33.9 |  |
| Secondary+ | 16.9 | 14.7 |  |
| Quintiles of wealth index |  |  |  |
| Poorest | 31.1 | 18.3 | <0.001 |
| Poorer | 23.7 | 19.6 |  |
| Middle | 17.9 | 21.1 |  |
| Richer | 17.6 | 20.4 |  |
| Richest | 9.8 | 20.7 |  |
| Urban residence (%) | 29.4 | 37.3 | <0.001 |
| Province |  |  |  |
| Balochistan | 4.7 | 10.6 | <0.001 |
| Khyber Pakhtunkhwa | 9.8 | 17.7 |  |
| Punjab | 13.2 | 56.2 |  |
| Sindh | 72.4 | 15.4 |  |

Unit of analysis in this table is mothers. This table shows that FALAH districts were significantly different from non-FALAH districts before the FALAH project started.
